# The role of calretinin-expressing granule cells in olfactory bulb functions and odor behavior

**DOI:** 10.1038/s41598-018-27692-8

**Published:** 2018-06-20

**Authors:** Delphine Hardy, Sarah Malvaut, Vincent Breton-Provencher, Armen Saghatelyan

**Affiliations:** 1 CERVO Brain Research Center, Quebec City, QC G1J 2G3 Canada; 2 Departement of Psychiatry and Neuroscience, Université Laval, Quebec City, QC G1K 7P4 Canada

## Abstract

The adult mouse olfactory bulb is continuously supplied with new neurons that mostly differentiate into granule cells (GCs). Different subtypes of adult-born GCs have been identified, but their maturational profiles and their roles in bulbar network functioning and odor behavior remain elusive. It is also not known whether the same subpopulations of GCs born during early postnatal life (early-born) or during adulthood (adult-born) differ in their morpho-functional properties. Here, we show that adult-born calretinin-expressing (CR^+^) and non-expressing (CR^−^) GCs, as well as early-born CR^+^ GCs, display distinct inhibitory inputs but indistinguishable excitatory inputs and similar morphological characteristics. The frequencies of inhibitory post-synaptic currents were lower in early-born and adult-born CR^+^ GCs than in adult-born CR^−^ neurons. These findings were corroborated by the reduced density of gephyrin^+^ puncta on CR^+^ GCs. CR^+^ GCs displayed a higher level of activation following olfactory tasks based on odor discrimination, as determined by an immediate early gene expression analysis. Pharmacogenetic inhibition of CR^+^ GCs diminished the ability of the mice to discriminate complex odor mixtures. Altogether, our results indicate that distinct inhibitory inputs are received by adult-born CR^+^ and CR^−^ GCs, that early- and adult-born CR^+^ neurons have similar morpho-functional properties, and that CR^+^ GCs are involved in complex odor discrimination tasks.

## Introduction

Granule cells (GCs) are the most abundant neuronal population in the olfactory bulb (OB). They make reciprocal dendro-dendritic synapses with mitral cells (MCs) and tufted cells (TCs)^1^ and are involved in the fine spatio-temporal tuning of the responses of these principal OB neurons to odors^2–4^. GCs receive glutamatergic inputs from MC/TCs and top-down fibers^5,6^ and inhibitory inputs from short-axon and Blanes cells^7,8^. The disinhibition of GCs^9^ and the pharmacogenetic and/or optogenetic manipulation of their activity^10,11^ affects the ability of mice to discriminate chemically similar odors and odor mixtures.

GCs are continuously renewed throughout life. During adulthood, neuronal precursors born in the subventricular zone (SVZ) of the lateral ventricle migrate along the rostral migratory stream (RMS) and, once in the OB, differentiate into inhibitory interneurons and integrate into the already functioning bulbar network^12–14^. Adult-born GC-mediated inhibition is required for the maintenance of the synchronized activity of MCs^15^. Environmental, pharmacological, optogenetic, and genetic manipulations that affect the number or activity of adult-born neurons modulate short-term odor memory^15,16^, olfactory perceptual learning^17^, fine olfactory discrimination^18,19^, and innate olfactory responses^20^. While these studies have considered GCs as a homogenous population of neurons, different GC subtypes have been identified based on their localization pattern and their expression of calretinin (CR)^21^, metabotropic glutamate receptor 2 (mGluR2)^22^, Ca^2+^/calmodulin-dependent protein kinase II α (CaMKIIα) and IV (CaMKIV)^23^, and glycoprotein 5T4^24^. The roles of these distinct GC subpopulations in OB network functioning and odor behavior may be quite different, depending on the level of activation of the cells by a given behavioral paradigm and their roles in the bulbar network. It is also unclear whether there are any morpho-functional differences between the same subpopulation of GCs born during early postnatal life or during adulthood. To start addressing these issues, we studied the integration and functional roles of CR-expressing and non-expressing (CR^+^ and CR^−^, respectively) adult-born and CR^+^ early-born GCs in the OB.

We showed that early-born CR^+^ and adult-born CR^+^ and CR^−^ GCs display similar dendritic arborization and spine density on secondary dendrites, and receive the same excitatory inputs. However, the frequencies of inhibitory post-synaptic currents were lower in both early-born and adult-born CR^+^ GCs than in adult-born CR^−^ GCs. In agreement with these functional results, an immunohistochemical analysis showed that there are fewer gephyrin^+^ puncta on the primary dendrites of adult-born CR^+^ GCs. We then assessed the involvement of CR^+^ GCs in different odor behavior tasks by co-immunolabeling for CR and the immediate early genes *cFos* and *Zif268*. Our results showed that CR^+^ GCs are involved in olfactory behaviors based on odor discrimination and that the pharmacogenetic inhibition of CR^+^ GCs results in a significant decrease in the fine odor discrimination ability of mice. Our results, thus, show that distinct inhibitory inputs impinge on adult-born CR^+^ and CR^−^ GCs, that early-born and adult-born CR^+^ GCs have similar morpho-functional properties, and that the CR^+^ GC subtype makes an important contribution to fine odor discrimination.

## Results

### CR^+^ and CR^−^ GCs display similar morphological characteristics

**Figure 1:**
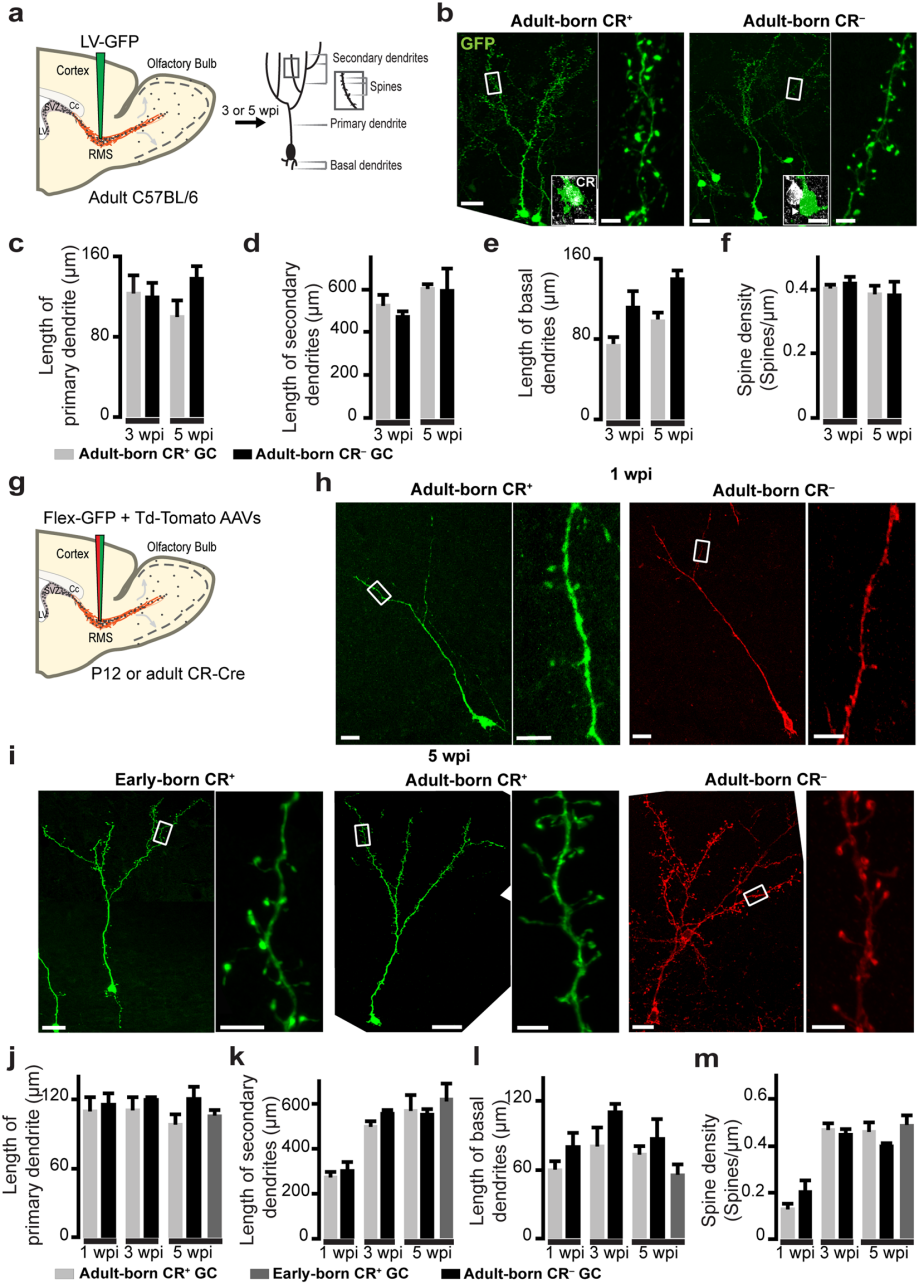
Morphological characterization of early-born and adult-born CR^+^ GCs and adult-born CR^−^ GCs (**a)** Schematic diagram showing the injection of GFP-encoding lentivirus into the RMS of adult mice and the morphometric parameters used to analyze the structural properties of CR^+^ and CR^−^ GCs. (**b)** Representative images of CR^+^ and CR^−^ GCs at three wpi. Insets show CR immunolabeling (white). Scale bars: 20 µm (left image), 4 µm (right image), and 10 µm (inset). (**c**–**f)** The mean lengths of the primary dendrite (**c**), secondary dendrites (**d**), and basal dendrites (**e**), and the spine densities (**f**) of adult-born CR^+^ and CR^−^ GCs at three and five wpi. (**g)** Schematic diagram showing the co-injection of the Flex-GFP and Tdtomato AAVs into the RMS of adult and P12 CR-Cre mice. (**h)** Example of adult-born CR^+^ and CR^−^ GCs at one wpi. (**i)** Examples of early-born and adult-born CR^+^ GCs and adult-born CR^−^ GCs at five wpi. Scale bars: 20 µm (left image) and 2 µm (right image). (**j**–**m)** The mean lengths of the primary dendrites (**j**), secondary dendrites (**k**), basal dendrites (**l**), and spine densities (**m**) of early-born and adult-born CR^+^ and adult-born CR^−^ GCs at one, three and five wpi. See Table 1.

Two different approaches were used to assess the morphological properties of adult-born CR^+^ and CR^−^ GCs. First, a GFP-encoding lentivirus was injected into the RMS of adult C57Bl/6 mice, and OB sections were immunolabeled for CR at three and five weeks post-injection (wpi) (Fig. 1a,b**)**. The pattern of localization of CR^+^ GCs in the granule cell layer (GCL) was not uniform and displayed a higher density in the superficial part of the GCL, which is why we decided to focus our study on GCs in that region (up to 200 µm from the mitral cell layer (MCL)). The mean lengths of the primary, secondary, and basal dendrites were then measured, and the average spine densities of the secondary dendrites of CR^+^ and CR^−^ virally labeled GCs in the superficial GCL were determined (Fig. 1c–f). No differences in these parameters were observed between the two subtypes of GCs at three and five wpi (Fig. 1b–f; Table 1**)**. At three wpi, the CR^+^ and CR^−^ adult-born GCs had fully developed primary and secondary dendrites that did not grow any further (Fig. 1c,d; Table 1). This was in line with our previous observations that GCs reach maturity at three wpi following the injection of viral vectors into the RMS^25^. There was also no significant difference in the mean length of the basal dendrites, although a tendency towards higher values in CR^−^ GCs was observed at both three and five wpi (Fig. 1e; Table 1). The spine densities of the secondary dendrites of the adult-born CR^+^ and CR^−^ GCs were also indistinguishable at three and five wpi (Fig. 1f; Table 1).

**Table 1:** Morphological properties of CR^+^ and CR^−^ GCs.

	Adult-born										Early-born
	C57BL/6 (LV-GFP + CR immunostaining)				CR-Cre + AAVs (Flex-GFP + Td-Tomato)						
	Three wpi		Five wpi		One wpi		Three wpi		Five wpi		
	CR^+^	CR^−^	CR^+^	CR^−^	CR^+^	CR^−^	CR^+^	CR^−^	CR^+^	CR^−^	CR^+^
Primary dendrites (µm)	124.70 ± 17.32	120.66 ± 12.82	101.26 ± 14.21	139.29 ± 10.98	112.24 ± 10.27	117.62 ± 8.51	113.00 ± 9.60	122.22 ± 1.00	100.61 ± 5.60	122.90 ± 9.52	108.03 ± 3.58
Secondary dendrites (µm)	531.46 ± 44.85	505.38 ± 22.33	611.31 ± 14.33	601.90 ± 96.42	276.80 ± 18.80	306.33 ± 30.90	507.23 ± 18.67	560.20 ± 9.87	572.03 ± 62.93	556.90 ± 17.90	625.22 ± 69.72
Basal dendrites (µm)	74.51 ± 7.68	112.65 ± 15.24	99.00 ± 6.70	141.22 ± 6.54	61.31 ± 6.36	81.56 ± 10.97	82.02 ± 15.21	112.50 ± 5.63	74.77 ± 5.85	88.95 ± 15.55	56.60 ± 7.26
	paired *t*(2) = −3.56, *p* = 0.07		paired *t*(2) = −0.55, *p* = 0.25		paired *t*(2) = −2.01, *p* = 0.18		paired *t*(2) = −2.65, *p* = 0.11			unpaired *t*(4) = 0.88, *p* = 0.13	
									unpaired *t*(4) = 1.95, *p* = 0.12		
Spine density (no. of spines/µm)	0.40 ± 0.01	0.44 ± 0.01	0.39 ± 0.02	0.39 ± 0.04	0.13 ± 0.02	0.21 ± 0.04	0.47 ± 0.02	0.45 ± 0.02	0.46 ± 0.03	0.40 ± 0.00	0.49 ± 0.03
					paired *t*(2) = −1.50, *p* = 0.27					unpaired *t*(4) = 3.15 *p* = 0.08	
Number of cells/animal	20/3	38/3	22/3	32/3	43/3	41/3	40/3	35/3	41/3	39/3	42/3

While this approach makes it possible to compare the structural properties of adult-born CR^+^ and CR^−^ GCs in fixed brain samples, it cannot be used to identify and functionally characterize these two subtypes in acute brain slices. We thus used a second approach to characterize the morpho-functional properties of CR^+^ and CR^−^ GCs. A mixture of Cre-dependent and Cre-independent AAVs driving GFP and tdTomato expression, respectively, were co-injected into the RMS of adult CalretininCre (CR-Cre) mice (Fig. 1g**)**. The maturational profiles of these cells at one, three, and five wpi were first analyzed. No differences in the mean lengths of the primary, secondary, and basal dendrites, or the average spine densities of the secondary dendrites of adult-born CR^+^ and CR^−^ GCs in the superficial GCL at one, three, or five wpi, were observed (Fig. 1h–m; Table 1). Adult-born CR^+^ and CR^−^ GCs displayed an immature morphology, with short secondary dendrites and few spines at one wpi (Fig. 1h,k and m; Table 1). They had, however, fully formed primary dendrites that had not grown any further at three wpi and five wpi (Fig. 1h–j; Table 1). This is consistent with observations that basal and primary dendrites start to develop rapidly as soon as adult-born GCs reach the GCL and that developing GCs receive their first synaptic inputs on their basal dendrites^6,12^. The main changes in the maturational profiles of adult-born CR^+^ and CR^−^ GCs occurred between one and three wpi and manifested as a two–fold increase in the length of the secondary dendrites ([F(2,6) = 15.5, *p* = 0.04, one-way ANOVA followed by a Bonferroni post-hoc test for CR^+^ GCs, *p* = 0.02; F(2,6) = 46.3, *p* = 0.0002, one-way ANOVA followed by a Bonferroni post hoc test for CR^−^ GCs, *p* = 0.0004]; Fig. 1k; Table 1) and in spine densities ([F(2,6) = 19.8, *p* = 0.01, one-way ANOVA followed by a Bonferroni post hoc test for CR^+^ GCs, *p* = 0.01; F(2,6) = 30.6, *p* = 0.0007, one-way ANOVA followed by a Bonferroni post hoc test for CR^−^ GCs, *p* = 0.0009]; Fig. 1m; Table 1). These changes were not, however, subtype-specific and were the same for CR^+^ and CR^−^ GCs. CR^+^ and CR^−^ GCs already had a mature morphology at three wpi that was very similar to that observed at five wpi in terms of the lengths of their primary and secondary dendrites and their spine densities (Fig. 1; Table 1). Two different approaches thus show that the maturational profiles of adult-born CR^+^ and CR^−^ GCs are indistinguishable.

To determine whether the structural properties of CR^+^ GCs differ depending on the developmental period during which they are generated, the primary, secondary, and basal dendrites as well as the spine densities of CR^+^ early-born and adult-born GCs were measured at five wpi. To compare the properties of fully mature early-born CR^+^ GCs with those of adult-born CR^+^ neurons, the Cre-dependent AAV was injected into the RMS of P12 CR-Cre mice, and their morphologies in the OB were examined at five wpi (Fig. 1g). No differences in their morphological parameters were observed (Fig. 1i–m; Table 1**)**.

These results suggest that superficial GCs have the same morphological characteristics independently of their phenotype (CR^+^ *vs*. CR^−^) and the developmental period during which they are generated (early-born CR^+^ *vs*. adult-born CR^+^ GCs).

### CR^+^ and CR^−^ GCs receive similar excitatory but different inhibitory inputs

**Figure 2:**
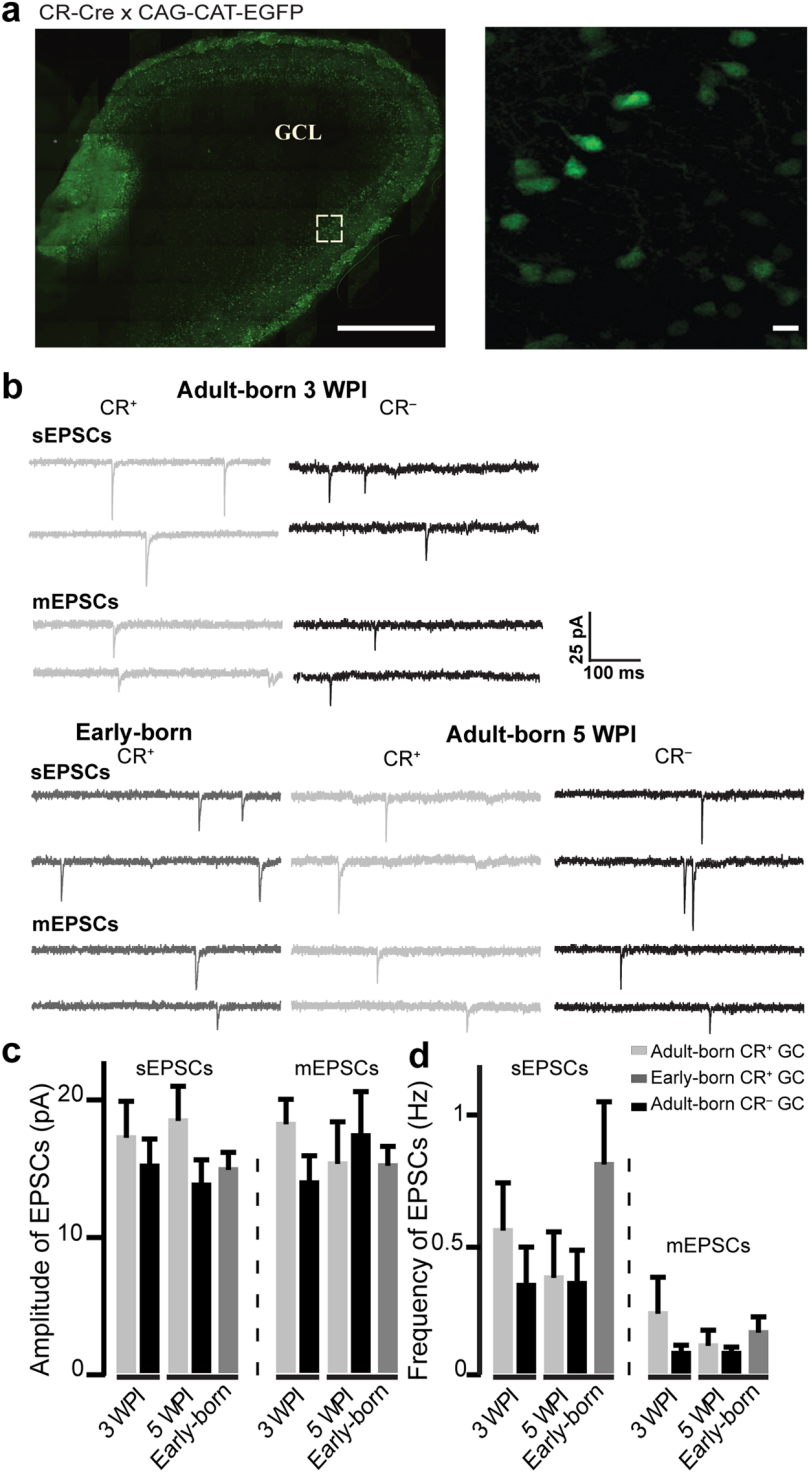
The excitatory postsynaptic inputs of CR^+^ and CR^−^ GCs are indistinguishable (**a)**. Labeling of early-born CR^+^ GCs in CR-Cre::CAG-CAT-GFP mice. The inset shows a higher magnification of GFP^+^ GCs in the GCL. Scale bars: 500 µm (left image) and 10 µm (right image). (**b)** Examples of sEPSC and mEPSC events recorded from early-born CR^+^ GCs, adult-born CR^+^ GCs, and CR^−^ GCs. (**c**,**d)** The mean amplitudes (**c**) and frequencies (**d**) of sEPSCs and mEPSCs recorded from early and adult-born CR^+^ GCs and adult-born CR^−^ GCs are indistinguishable. See Table 3.

We next determined whether CR^+^ and CR^−^ GCs are functionally distinct and have different passive and/or active membrane properties or receive different excitatory and/or inhibitory inputs. The same labeling strategy described above (co-injection of Cre-dependent and Cre-independent GFP and tdTomato AAVs into CR-Cre mice) was used to compare postsynaptic currents in adult-born CR^+^ and CR^−^ GCs. Whole-cell patch-clamp recordings were taken at three and five wpi in acute OB slices. To target early-born CR^+^ GCs, another transgenic mouse line obtained from the cross-breeding of CR-Cre mice with GFP reporter mice (CAG-CAT-GFP), in which all CR^+^ cells express GFP, was used (Fig. 2a). It has been previously shown that adult-born neurons make up only 10–15% of the entire populations of GCs^26,27^. As such, the vast majority of GFP^+^ cells in this mouse line are early-born CR^+^ GCs.

We first measured intrinsic membrane properties of adult-born CR^+^ and CR^−^ GCs at three and five wpi, as well as early-born CR^+^ GCs. No differences in the resting membrane potential (Vm), the membrane resistance (Rm), the capacitance (Cm), as well as the activation threshold of voltage-gated Na^+^ current (I_Na+_) and its peak amplitude were observed (Table 2). We next recorded action potential-dependent and independent spontaneous excitatory and inhibitory events from adult-born CR^+^ and CR^−^ GCs at three and five wpi, as well as early-born CR^+^ GCs. GCs receive excitatory inputs from MC/TCs and centrifugal fibers^6,28^ and inhibitory inputs from short-axon and Blanes cells^8^. To record spontaneous excitatory postsynaptic currents (sEPSCs), GABA_A_ receptors were blocked by the bath application of bicuculline methochlorine (BMI). No differences in sEPSC amplitudes or frequencies were observed between adult-born CR^+^ and CR^−^ GCs at three wpi (Fig. 2b–d; Table 3). An analysis of action potential-independent miniature EPSCs (mEPSCs) isolated by the application of tetrodotoxin (TTX) also showed that the two sub-populations of adult-born GCs have similar mEPSC amplitudes and frequencies (Fig. 2b–d; Table 3). To determine whether functional differences between adult-born CR^+^ and CR^−^ GCs may arise at later time-points, patch-clamp recordings were also performed at five wpi. Again, no differences between CR^+^ and CR^−^ adult-born GCs were observed in terms of the amplitudes and frequencies of their sEPSCs and mEPSCs (Fig. 2b–d; Table 3). These results suggest that excitatory inputs impinging on CR^+^ and CR^−^ GCs are indistinguishable. To determine whether there are any differences in the same population of GCs that are generated during different stages of life, sEPSCs and mEPSCs were also recorded from early-born CR^+^ GCs and were compared to those recorded from adult-born CR^+^ neurons. sEPSCs recorded from early-born CR^+^ GCs were similar to those of adult-born CR^+^ cells at five wpi, although a difference, albeit not significant, was observed in the frequencies of sEPSCs (Fig. 2b–d; Table 3). Similarly, the mEPSC amplitudes and frequencies of early-born and adult-born CR^+^ GCs were comparable **(**Fig. 2b–d; Table 3). These electrophysiological results are in line with the similar spine densities of the secondary dendrites observed with adult-born CR^+^ and CR^−^ GCs and early-born CR^+^ neurons (Fig. 1).

**Table 2:** Intrinsic properties of early- and adult-born CR^+^ GCs and adult-born CR^−^ GCs.

	Adult-born				Early-born
	Three wpi		Five wpi		
	CR^+^	CR^−^	CR^+^	CR^−^	CR^+^
Vm (mV)	−64.78 ± 1.73	−65.38 ± 2.69	−62.25 ± 3.90	−64.67 ± 2.72	−63.50 ± 2.65
Rm (GΩ)	1.14 ± 0.06	1.16 ± 0.09	1.06 ± 0.09	0.97 ± 0.10	1.10 ± 0.15
Cm (pF)	9.29 ± 1.53	11.48 ± 1.34	10.38 ± 1.28	10.11 ± 0.77	8.15 ± 0.59
I_Na_^+^ threshold (mV)	−35.56 ± 4.75	−38.89 ± 2.00	−32.50 ± 10.13	−38.75 ± 2.27	−37.78 ± 1.47
I_Na_^+^ peak of amplitude (nA)	0.731 ± 0.093	1.135 ± 0.155	0.961 ± 0.212	1.091 ± 0.196	0.813 ± 0.046
	*U*(16) = 17, *p* = 0.07				
Number of cells/animal	10/7	9/6	8/6	9/5	11/6

**Table 3:** EPSC amplitudes and frequencies of early-born and adult-born CR^+^ GCs and adult-born CR^−^ GCs.

		Adult-born				Early-born
		Three wpi		Five wpi		
		CR^+^	CR^−^	CR^+^	CR^−^	CR^+^
sEPSCs	Amplitude (pA)	17.33 ± 2.50	15.23 ± 1.85	18.60 ± 2.33	13.83 ± 1.74	14.98 ± 1.11
				*U*(15) = 21, *p* = 0.16		
	Frequency (Hz)	0.56 ± 0.18	0.35 ± 0.14	0.38 ± 0.17	0.36 ± 0.14	0.82 ± 0.23
		*U*(17) = 35, *p* = 0.43			*U*(18) = 23.5, *p* = 0.052	
				*U*(17) = 25.5, *p* = 0.13		
	Number of cells/animal	10/7	9/6	8/6	8/5	11/6
mEPSCs	Amplitude (pA)	18.32 ± 1.62	13.98 ± 1.80	15.40 ± 2.92	17.51 ± 2.98	15.27 ± 1.18
		*U*(15) = 19, *p* = 0.11				
	Frequency (Hz)	0.24 ± 0.13	0.09 ± 0.02	0.12 ± 0.05	0.09 ± 0.03	0.17 ± 0.05
		*U*(15) = 32, p = 0.72				
	Number of cells /animals	8/7	9/6	8/6	9/6	10/5

**Figure 3:**
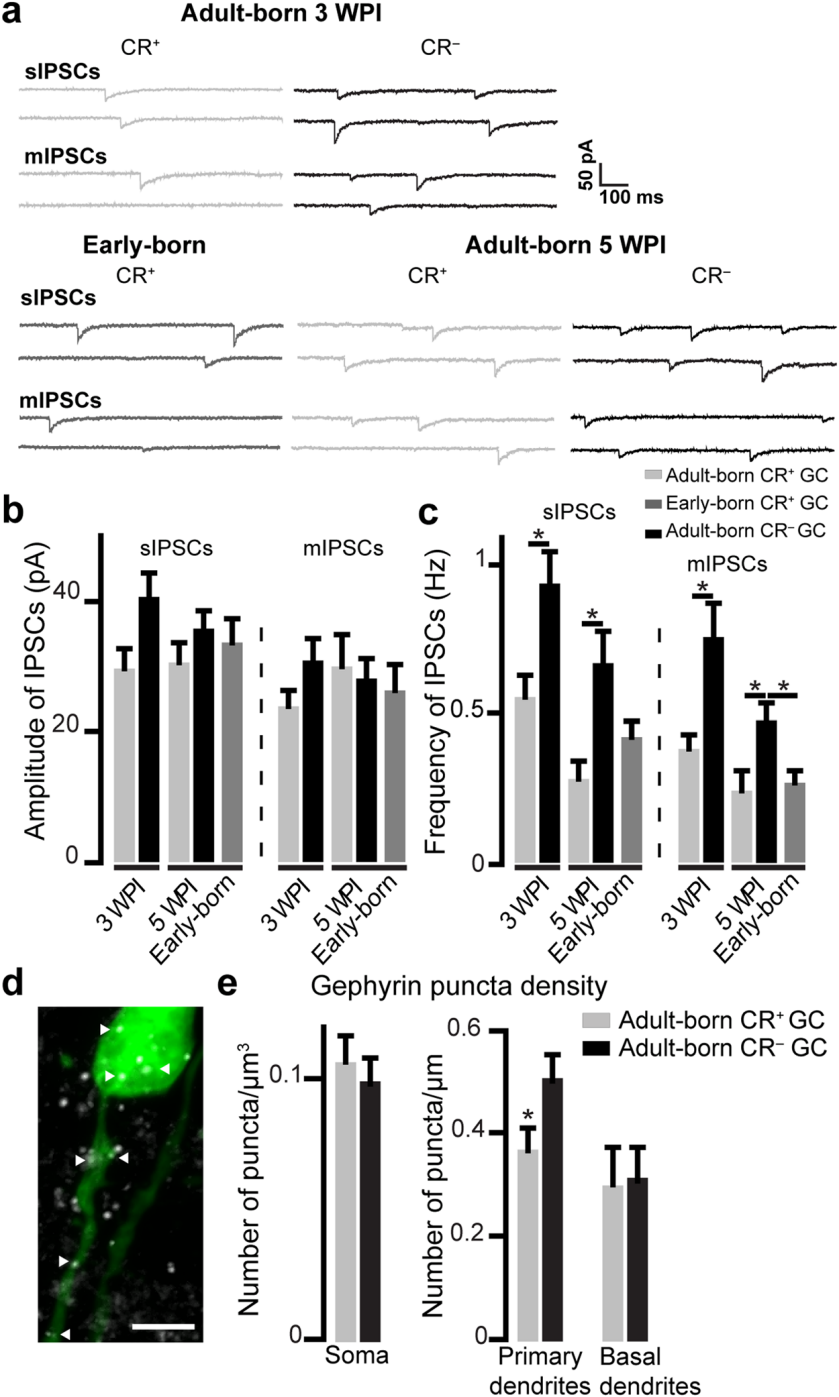
CR^+^ GCs receive weaker inhibitory inputs than CR^−^ GCs and have fewer gephyrin^+^ puncta on their primary dendrites (**a)**. Examples of sIPSCs and mIPSCs recorded from early-born CR^+^ GCs, and adult-born CR^+^ and CR^−^ CGs. (**b**,**c)**. Mean amplitudes (**b**) and frequencies (**c**) of sIPSCs and mIPSCs recorded from early and adult-born CR^+^ GCs and adult-born CR^−^ GCs. See Table 4. (**d)**. Representative image of virally labeled GCs (green) that have also been labeled for gephyrin (white). Arrowheads indicate gephyrin^+^ puncta. Scale bars: 10 µm. (**e)**. Mean densities of gephyrin^+^ puncta on the primary and basal dendrites and cell soma of CR^+^ and CR^−^ adult-born GCs at three wpi. See Table 4. *p < 0.05 Mann-Whitney *U*-test for IPSCs frequencies and paired *t*-test for gephyrin analysis.

Inhibitory postsynaptic currents (IPSCs) isolated by the bath application of kynurenic acid (Kyn) were also recorded. As with EPSCs, no differences in the amplitudes of sIPSCs and mIPSCs were noted between adult-born CR^+^ and CR^−^ GCs at three and five wpi (Fig. 3a,b; Table 4). In contrast, we observed that the frequencies of sIPSCs and mIPSCs in CR^+^ GCs are lower than those in CR^−^ GCs at both three and five wpi (three wpi, sIPSCs: 0.56 ± 0.07 Hz *vs*. 0.94 ± 0.11 Hz for CR^+^ and CR^−^ GCs, respectively, *p* = 0.008, Mann-Whitney *U* test; three wpi, mIPSCs 0.38 ± 0.05 Hz *vs*. 0.76 ± 0.11 Hz for CR^+^ and CR^−^ GCs, respectively, *p* = 0.002, Mann-Whitney *U* test; five wpi, sIPSCs 0.28 ± 0.06 Hz *vs*. 0.67 ± 0.11 Hz for CR^+^ and CR^−^ GCs, respectively, *p* = 0.007, Mann-Whitney *U* test; and five wpi, mIPSCs 0.24 ± 0.07 *vs*. 0.47 ± 0.07 for CR^+^ and CR^−^ GCs, respectively, *p* = 0.02, Mann-Whitney *U* test Fig. 3a–c; Table 4**)**.

**Table 4:** IPSC amplitudes and frequencies of early- and adult-born CR^+^ GCs and adult-born CR^−^ GCs, and number of gephyrin immunopuncta on adult-born CR^+^ and CR^−^ GCs.

		Adult-born				Early-born
		Three wpi		Five wpi		
		CR^+^	CR^−^	CR^+^	CR^−^	CR^+^
sIPSCs	Amplitude (pA)	29.52 ± 3.15	40.60 ± 3.73	30.41 ± 3.15	35.56 ± 2.97	33.40 ± 3.92
		*U*(22) = 39.5, *p* = 0.06				
	Frequency (Hz)	0.56 ± 0.07	0.94 ± 0.11	0.28 ± 0.06	0.67 ± 0.11	0.42 ± 0.06
		*U(22) = 27.5, *p* = 0.008		*U(19) = 18, *p* = 0.007		
					*U*(18) = 27, *p* = 0.09	
				*U*(19) = 31, *p* = 0.09		
	Number of cells/animal	13/8	11/5	11/6	10/5	10/4
mIPSCs	Amplitude (pA)	23.83 ± 2.51	30.86 ± 3.24	29.96 ± 4.77	28.08 ± 3.10	26.25 ± 3.90
		*U*(20) = 40, *p* = 0.23				
	Frequency (Hz)	0.38 ± 0.05	0.76 ± 0.11	0.24 ± 0.07	0.47 ± 0.07	0.27 ± 0.04
		*U(20) = 15, *p* = 0.002		*U(17) = 17, *p* = 0.02		
					*U(18) = 23, *p* = 0.04	
	Number of cells/animal	13/8	9/4	9/5	10/5	10/4
Gephyrin immunopuncta density	Soma (nb. of puncta/µm³)	0.11 ± 0.01	0.10 ± 0.01			
	Basal (nb. of puncta/µm²)	0.30 ± 0.07	0.31 ± 0.06			
	Primary (nb. of puncta/µm²)	0.37 ± 0.04	0.51 ± 0.04			
		*paired *t*(2) = 10.1, *p* = 0.01				

To provide further evidence that adult-born CR^+^ and CR^−^ GCs receive distinct inhibitory inputs, we performed gephyrin immunolabeling and determined the densities of gephyrin^+^ puncta on cell bodies, the proximal part of primary dendrites (up to 50 µm), and the basal dendrites of adult-born CR^+^ and CR^−^ GCs in the superficial GCL. While no differences in gephyrin puncta density on the cell bodies and basal dendrites of these two subtypes of GCs were observed, the density of gephyrin^+^ puncta on CR^−^ GC primary dendrites was higher than those of CR^+^ GCs (0.37 ± 0.04 and 0.51 ± 0.04 mean ± SE for CR^+^ and CR^−^ GCs respectively, [*t*(4) = 10.1, *p* = 0.01, paired *t*-test]; Fig. 3d,e; Table 4). These results are in line with the electrophysiological results and show that CR^+^ GCs have fewer inhibitory synapses on their primary dendrites than CR^−^ neurons, resulting in a lower level of inhibition.

To determine whether adult-born CR^+^ GCs receive different inhibitory inputs than their early-born counterparts, sIPSCs and mIPSCs were also recorded from early-born CR^+^ GCs. As with EPSCs, there were no differences in the amplitudes and frequencies of sIPSCs and mIPSCs recorded from early- and adult-born CR^+^ GCs at five wpi (Fig. 3a–c; Table 4). However, the frequencies of mIPSCs recorded from early-born CR^+^ GCs were significantly lower than those recorded from adult-born CR^−^ GCs at five wpi (0.27 ± 0.04 Hz *vs*. 0.47 ± 0.07 Hz for early-born CR^+^ and adult-born CR^−^ at five wpi, respectively, p = 0.043, Mann-Whitney *U* test]; Fig. 3a,c; Table 4). Altogether, these results show that although early- and adult-born CR^+^ GCs receive similar excitatory and inhibitory inputs, adult-born CR^+^ and CR^−^ GCs in the superficial GCL receive similar excitatory but different inhibitory inputs.

### Spontaneous odor discrimination increases the activation of CR^+^ GCs

To understand the involvement of CR^+^ GCs in olfactory behavioral tasks, the cells were immunolabeled for CR as well as the immediate early genes (IEG) *cFos* and *Zif268*, which are commonly used as cell activity markers^29–31^. The involvement of CR^+^ GCs in odor discrimination tasks was tested as superficial GCs may preferentially contact TCs^32–34^, which have been shown to synchronize the activity of iso-functional glomeruli and thus the odor discrimination ability of animals^35^. Given that no morphological or electrophysiological differences were observed between early-born and adult-born CR^+^ GCs, the analysis was performed on the total population of CR^+^ GCs, regardless of whether they were produced during early development or adulthood.

**Figure 4:**
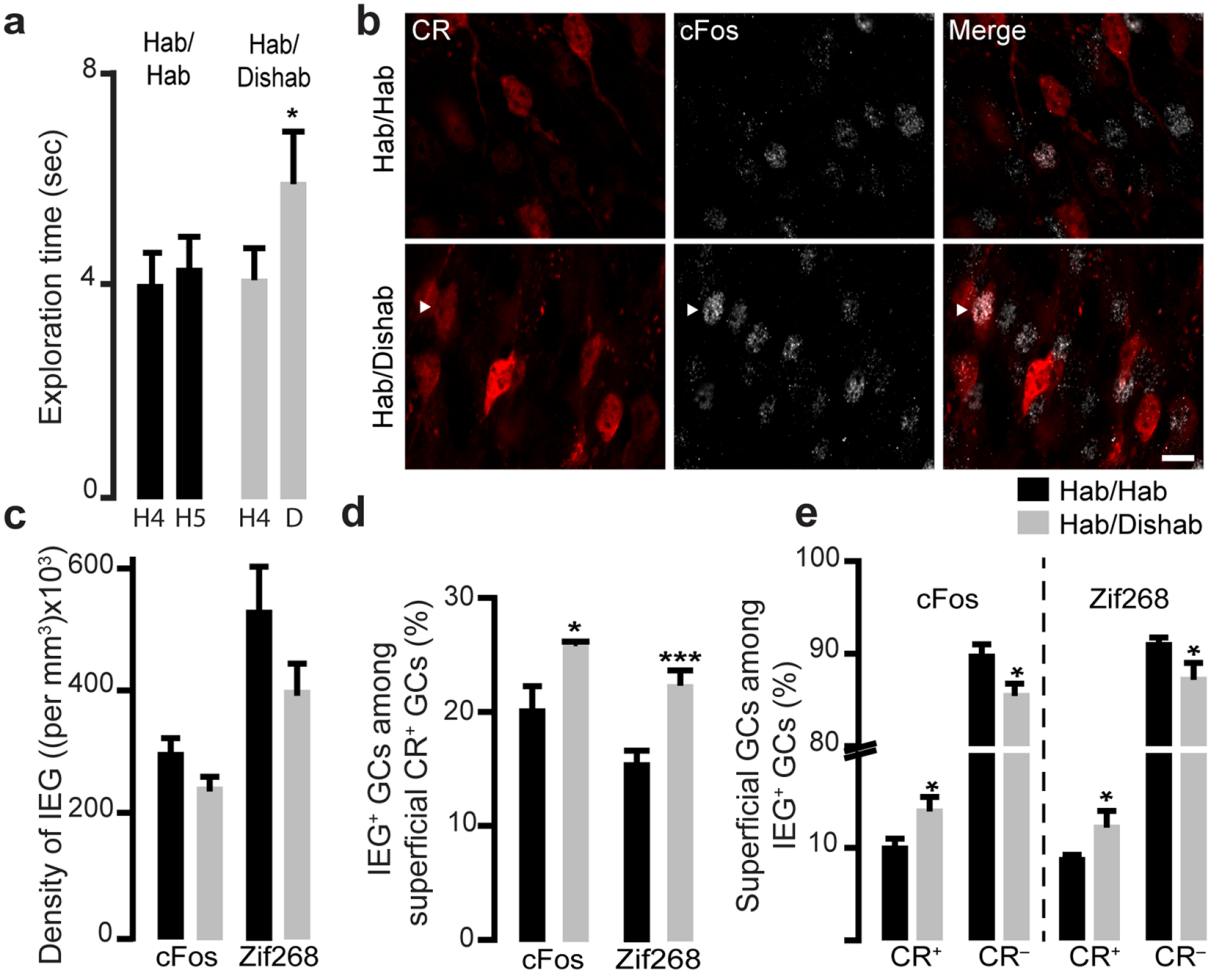
Spontaneous odor discrimination induces the activation of CR^+^ GCs (**a)**. Spontaneous odor discrimination task based on odor habituation/dishabituation (hab/dishab). C57Bl/6 mice were habituated to the presence of a first odor (the habituation odor ((+)-carvone)), which was presented four times. Their ability to discriminate between two similar odors was then investigated by the presentation of a second odor (the dishabituation odor ((−)-carvone)), which was chemically similar to the habituation odor. As expected, mice correctly discriminated between the two odors as shown by the increase in exploration time when the dishabituation odor was presented compared to the fourth presentation of the habituation odor (a, right panel). We used mice that were not dishabituated and that received the habituation odor ((+)-carvone) during the last presentation (hab/hab) as a control (a, left panel). (**b)** Examples of CR and cFos immunolabeling in mice from the control and odor discrimination groups. Arrowheads indicate cFos^+^/CR^+^ GCs. Scale bar: 10 µm. (**c–e**) Quantification of the density of cFos^+^ and Zif268^+^ GCs (**c**), the percentages of cFos^+^ and Zif268^+^ GCs among CR^+^ GCs (**d**), and the percentage of CR^+^ (left panel) and CR^−^ (right panel) GCs among cFos^+^ and Zif268^+^ GCs (**e**). *p < 0.05, ***p < 0.001 paired *t*-test for behavioral task and unpaired *t*-test for IEG analysis.

We first used a spontaneous odor habituation/dishabituation task. As expected, the mice in the habituation/dishabituation group spent more time exploring the novel dishabituating odor than during the 4th habituation session (mean exploration time: 4.1 ± 0.5 s for the last habituation session (H4) and 5.9 ± 1.0 s for the dishabituation (D) odor, *n* = 10 mice, *t*(10) = −2.5, *p* = 0.03, paired *t*-test; Fig. 4a). This increase was not observed in the habituation/habituation group, where the mice were not dishabituated (mean exploration time: 4.0 ± 0.5 s for H4 *vs*. 4.3 ± 0.6 s for H5, *n* = eight mice, Fig. 4a). An analysis of the densities of cFos^+^ and Zif268^+^ GCs in the superficial GCL did not reveal any differences between the two groups (306,855 ± 23,485 cells/mm^3^ and 243,407 ± 18,507 cells/mm^3^ for cFos^+^ and 528,287 ± 77,365 cells/mm^3^ and 403,618 ± 47,760 cells/mm^3^ for Zif268^+^, for the habituation and dishabituation groups, respectively), suggesting that superficial GCs undergo a similar level of activation (Fig. 4b,c). We next determined whether the spontaneous odor discrimination task induces the activation of CR^+^ GCs by analyzing the percentage of cFos^+^ and Zif268^+^ cells among CR^+^ GCs (Fig. 4d), and assessed whether the level of activation of CR^+^ and CR^−^ GCs differs by analyzing the percentage of CR^+^ and CR^–^ GCs among the cFos^+^ and Zif268^+^ cells (Fig. 4e). An analysis of cFos^+^ cells among the CR^+^ GCs revealed that the odor dishabituation task induced a higher activation of CR^+^ GCs (% cFos^+^ among CR^+^ GCs was 26.1 ± 0.2%, *n* = 634 CR^+^ cells) than the odor habituation task (% cFos^+^ cells among CR^+^ GCs was 20.6 ± 1.9%, *n* = 713 CR^+^ cells) [*t*(4) = 2.8, *p* = 0.04, unpaired *t*-test] (Fig. 4b,d). The same results were obtained with Zif268 (22.6 ± 0.8% *vs*. 15.8 ± 0.5% for Zif268^+^ cells among CR^+^ cells following the odor dishabituation and habituation tasks, respectively, *n* = 1270 and 904 CR^+^ GCs,) [*t*(6) = −6.7, *p* = 0.0005, unpaired *t*-test] (Fig. 4d).

Although these results indicated that CR^+^ GCs are activated by the spontaneous odor discrimination task, they do not show whether the activation is higher in the CR^+^ subtype or whether it occurs in all superficial GCs, including CR^–^ GCs. We thus determined the percentage of CR^+^ GCs (and by consequence CR^–^ GCs) among cFos^+^ and Zif268^+^ cells. The percentage of CR^+^ GCs among cFos^+^ and Zif268^+^ cells also increased (14.2 ± 1.1% *vs*. 10.1 ± 1.0% for CR^+^ GCs among cFos^+^ GCs, *n* = 1228 and 1443 cFos^+^ cells for the dishabituation and habituation groups, respectively) [*t*(4) = 2.8, *p* = 0.04, unpaired *t*-test]; (12.4 ± 1.3% *vs*. 8.8 ± 0.4% for CR^+^ GCs among Zif268^+^ GCs, *n* = 2553 and 1653 Zif268^+^ cells) [*t*(6) = −2.47, *p* = 0.04, unpaired *t*-test] (Fig. 4e). These results show that there is greater CR^+^-specific activation of GCs following the spontaneous odor discrimination task.

### The go/no-go olfactory learning task induces the activation of CR^+^ GCs

**Figure 5:**
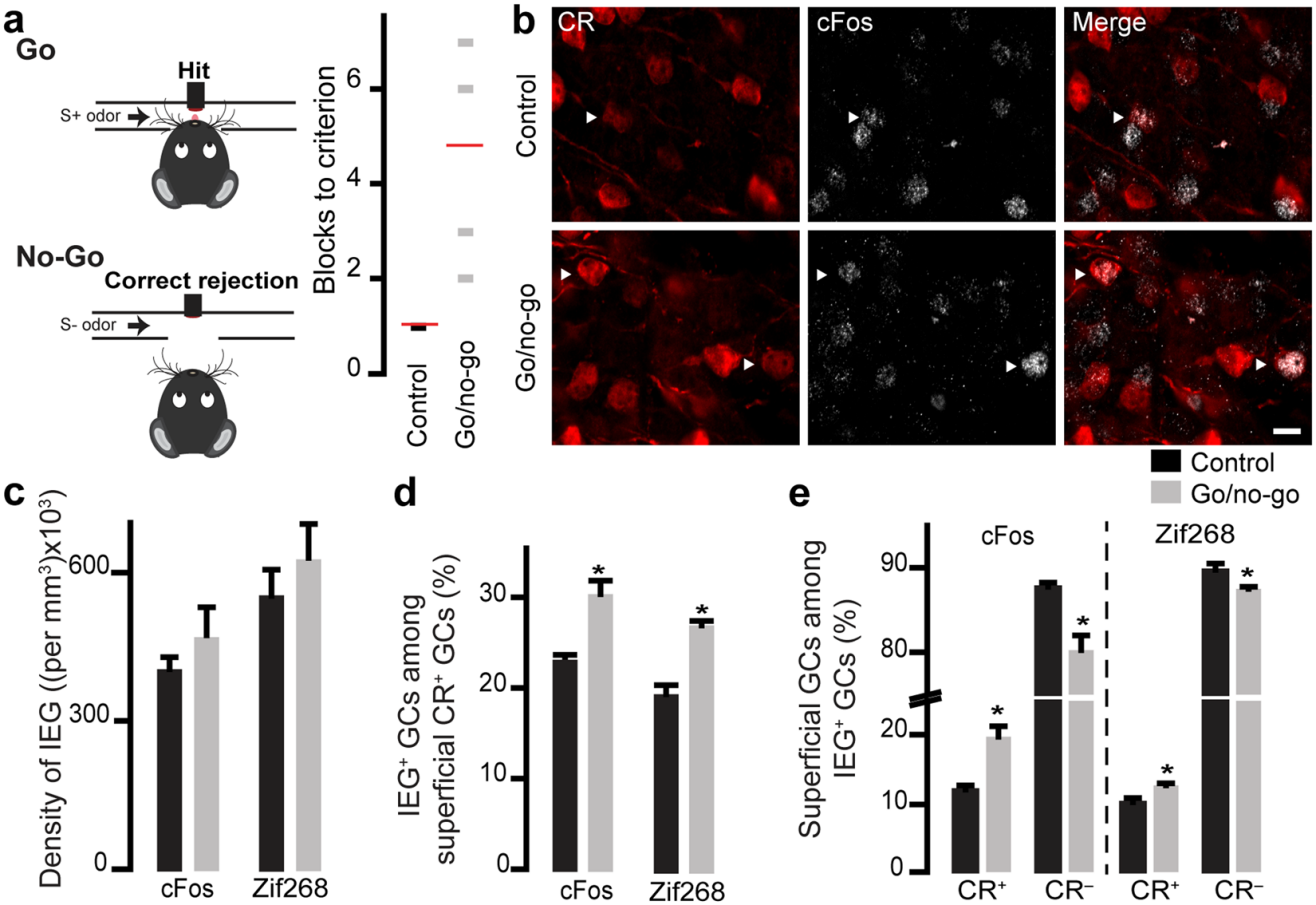
Odor discrimination learning activates CR^+^ GCs (**a)**. Water-restricted C57Bl/6 male mice were tested using the go/no-go odor discrimination task. Mice were randomly exposed to reward-associated and non-reward-associated odors (S+ and S−, respectively), and the percentage of correct responses (hits + correct rejections) was calculated for every 20 trials. The mice were considered to have successfully discriminated between the two odors if they reached the 85% criterion of correct responses. The odors used were 0.1% octanal and 0.1% decanal (odor pair 1). Number of blocks needed in each group to reach the 85% criterion (right panel). For mice in the control group, the water was given independently of the odor. (**b)**. Examples of immunolabeling for CR and cFos on OB slices after the go/no-go odor discrimination task. Arrowheads indicate cFos^+^/CR^+^ GCs. Scale bar: 10 µm. (**c-e**) Quantification of the densities of cFos^+^ and Zif268^+^ GCs (**c**), the percentages of cFos^+^ and Zif268^+^ GCs among CR^+^ GCs (**d**), and the percentages of CR^+^ (left panel) and CR^−^ (right panel) GCs among cFos^+^ and Zif268^+^ GCs (**e**). Note the higher percentages of CR^+^/cFos^+^ and CR^+^/Zif268^+^ GCs, indicating that CR^+^ GCs are specifically involved in odor discrimination. *p < 0.05 unpaired *t*-test.

Since the spontaneous discrimination task caused a significant increase in CR^+^ GC recruitment, we hypothesized that this subtype would be also activated by an operant go/no-go odor discrimination task. An olfactometer was used to train the mice to associate a stimulus odor (octanal: S+) with a water reward. When the S+ odor is presented, the mice should lick the water port and when a non-reward-associated odor (decanal: S−) is presented, the animals should not lick the water port. We considered that the mice were able to discriminate correctly between the two odors when they reached the 85% criterion of correct responses. For the control group, the water was presented independently of the S+ or S− odor. As expected, the control group reached the 85% criterion very rapidly (one block) whereas the experimental group needed more blocks to reach the criterion (mean of 4.8 blocks) (Fig. 5a). To avoid bias in the activation of GCs because of different levels of exposure of the control and experimental groups to the odors, the mice in the control group, like the mice in the experimental group (go/no-go), had to complete five blocks.

No differences in the densities of cFos^+^ and Zif268^+^ GCs were observed between the two groups (for cFos: 473,883 ± 47,932 cells/mm^3^ and 410,158 ± 24,965 cells/mm^3^, go/no-go and control groups, respectively; and for Zif268: 630,814 ± 59,064 cells/mm^3^ and 570,921 ± 44,985 cells/mm^3^ for the go/no-go and control groups, respectively; Fig. 5b,c). However, the percentage of activated cFos/CR^+^ GCs was higher after the go/no-go odor discrimination task. The mean percentages of cFos^+^ cells among CR^+^ GCs were 30.7 ± 1.7% *vs*. 23.5 ± 0.8% for the go/no-go and control groups, *n* = 1590 and 1167 cells, respectively, ([*t*(6) = −2.9, *p* = 0.02, unpaired *t*-test] Fig. 5d). The activation was higher for the CR^+^ sub-population of GCs since the percentage of CR^+^ GCs among cFos^+^ cells was significantly higher following go/no-go odor discrimination task, suggesting that there is a decrease in the percentage of CR^−^ GCs among cFos^+^ (the mean percentage of CR^+^ GCs among cFos^+^ cells was 19.7 ± 1.7% *vs*. 12.1 ± 0.4%, go/no-go and control groups, *n* = 2732 and 2255 cells, respectively; ([*t*(6) = −3.1, *p* = 0.02, unpaired *t*-test]; Fig. 5e). The same results were observed with Zif268. The mean percentages of Zif268^+^/CR^+^ GCs were 27.4 ± 2.1% *vs*. 19.7 ± 0.9%, *n* = 897 and 941 cells for the go/no-go and control groups, respectively; [*t*(6) = −2.6, *p* = 0.03, unpaired *t*-test]), and the mean percentages of CR^+^ GCs among Zif268^+^ GCs were 12.6 ± 0.4% *vs*. 10.2 ± 0.7% for the go/no-go and control groups, *n* = 1809 and 1820 cells, respectively ([*t*(6) = −3.2, *p* = 0.01, unpaired *t*-test]; Fig. 5d,e). These results suggest that CR^+^ GCs are recruited following the go/no-go odor discrimination task.

### The pharmacogenetic inhibition of CR^+^ GCs diminishes the fine olfactory discrimination ability of mice

**Figure 6:**
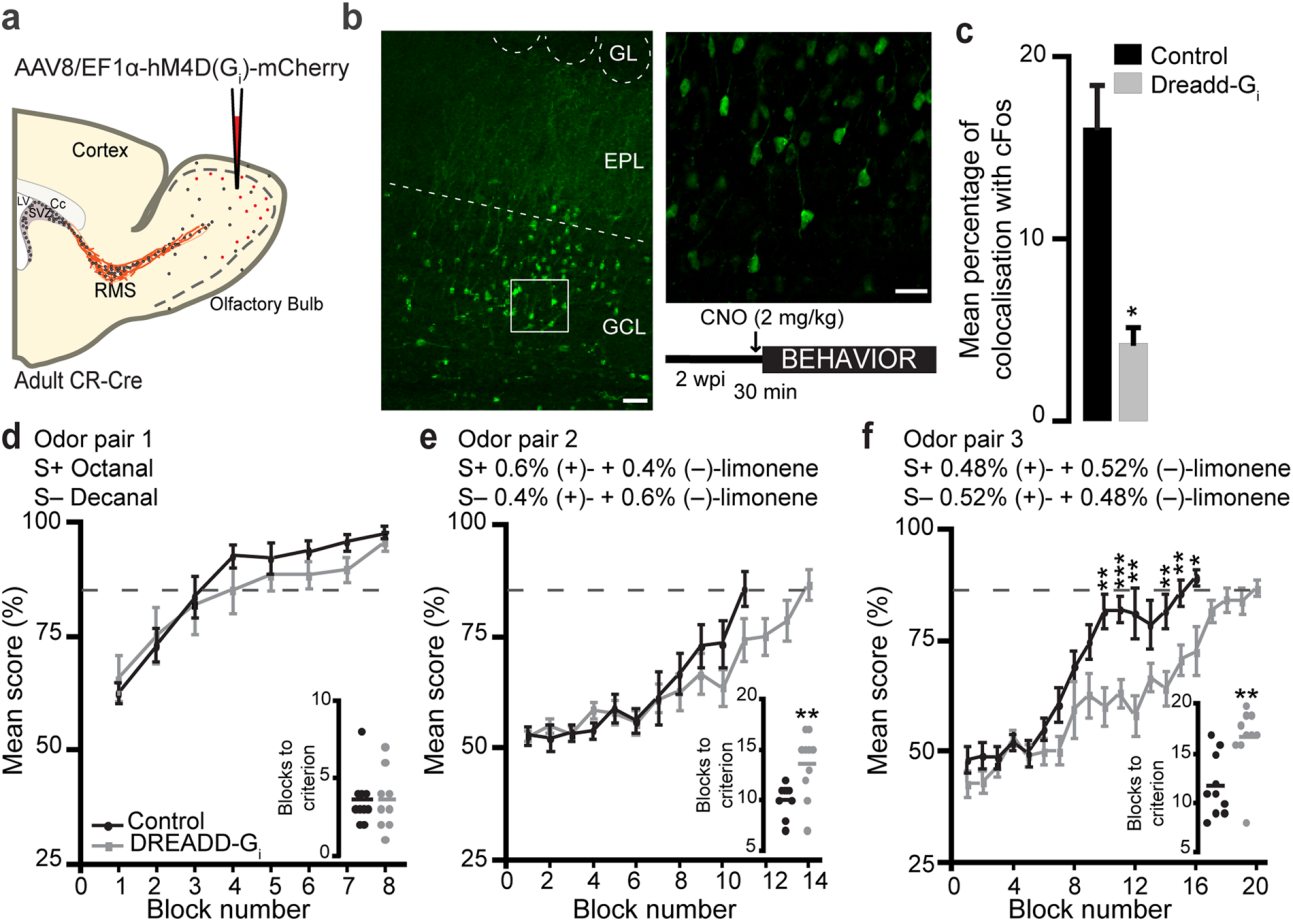
The pharmacogenetic inhibition of CR^+^ GCs reduces the olfactory discriminatory ability of mice (**a**). The EF1α-DIO-hM4D(G_i_)-mCherry AAV was injected into the OB of CR-Cre mice, inducing the expression of the G_i_-coupled DREADDs receptor by CR^+^ GCs. Control mice were injected with a Cre-independent GFP-encoding AAV, which labeled all GCs. The behavior experiments started at two wpi, and the mice in the two groups were given an intraperitoneal injection of 2 mg/kg of CNO 30 min prior undertaking the behavior task. (**b**) Example of the injection of the AAV in the OB. Scale bar: 50 µm. The inset shows a higher magnification image of DREADD-G_i_ infected cells. Scale bar: 10 µm. (**c**) Quantification of the percentages of reporter^+^ (GFP or mCherry) and cFos^+^ GCs following the CNO injections in mice previously injected with the control (EF1α-DIO-EYFP) or the DREADDs (EF1a-DIO-hM4D(G_i_)-mCherry) AAV. Note the effective pharmacogenetic inhibition of CR^+^ GCs (*p < 0.05 paired *t*-test). (**d**–**f**) The mean scores in percentages for each block of 20 trials obtained from the control and DREADDs AAV-injected mice for the go/no-go odor discrimination task using odor pairs of different complexity. Odor pair 1: 0.1% octanal (S+) *vs*. 0.1% decanal (S−), *n* = 10 and nine mice for the control and DREADDs groups, respectively (**d**); Odor pair 2: 0.6% (+)- + 0.4% (−)-limonene (S+) *vs*. 0.4% (+)- + 0.6% (−)-limonene (S−), *n* = nine and 10 mice for the control and DREADDs groups, respectively (**e**); Odor pair 3, 0.48% (+)- + 0.52% (−)-limonene (S+) *vs*. 0.52% (+)- + 0.48% (−)-limonene (S−), *n* = 10 mice for the control and DREADDs groups, respectively (**f**). The dashed lines represent the 85% criterion score. Insets show the mean values for each group to reach the criterion of 85% correct responses (*p < 0.05; **p < 0.01 unpaired *t*-test).

Double immunolabeling for CR and Zif268 or cFos showed that CR^+^ GCs are activated by the odor discrimination task. To determine whether CR^+^ GCs are required for accomplishment of the odor discrimination task, we used pharmacogenetic tools that specifically inhibit CR^+^ GCs and subjected the mice to the go/no-go odor discrimination task (Fig. 6a). The Cre-dependent designer receptors exclusively activated by designer drugs (DREADDs) coupled with a G_i_ protein AAV was injected into the GCL of the CR-Cre OB two weeks before beginning the behavioral task (Fig. 6a). DREADDs-G_i_ activation by CNO leads to GIRK-mediated K^+^ ion extrusion^36^ and hyperpolarization of the cell. The control group of mice was injected with AAV-GFP, and the two groups received an injection of clozapine-N-oxide (CNO) 30 min prior the task.

The percentage of infected cells among the CR^+^ GCs at the site of injection was 45.0 ± 11.0% (*n* = 403 cells from three mice), and we estimate that DREADDs-infected GCs represent about 8% (*n* = three mice) of the bulbar CR^+^ GCs. Almost all the DREADDs-infected cells were CR^+^ (92.8 ± 0.9%, *n* = 178 cells from three mice) and essentially no infected CR^+^ periglomerular cells were observed (3.6 ± 2.0%, *n* = 1731 cells from six mice). To test the efficiency of the pharmacogenetic inhibition, we analyzed the percentage of colocalization of reporter^+^ cells (GFP or mCherry) and cFos^+^ cells in the control (EF1α-DIO-EYFP) and DREADDs (EF1α-DIO-hM4D(G_i_)-mCherry) AAV-injected CR-Cre mice at three wpi. Significantly lower percentages of reporter^+^/cFos^+^ (thus CR^+^/cFos^+^) GCs were observed 90 min following the pharmacogenetic inhibition of the CR^+^ GCs (4.2 ± 0.8% *vs*. 16.0 ± 2.2% in the DREADDs-injected and control mice, *n* = 177 and 305 cells, respectively, [*t*(6) = −4.5, *p* = 0.02, paired *t*-test]; Fig. 6c).

We next subjected mice to go/no-go odor discrimination task using different odor combinations that progressively increased the difficulty of the task from odor pair 1 (Fig. 6d) to odor pair 3 (Fig. 6f). As expected, the number of blocks needed to reach the criterion of 85% of correct responses increased with the difficulty of the discrimination task for both the control and DREADDs groups. There were no differences between the mice in the two groups that were exposed to odor pair 1, and the two groups were equally successful in reaching the 85% criterion in one day (the mean number of blocks to reach the 85% criterion was 3.5 for the control group and 3.6 for the DREADDs group; *n* = nine and 10 mice, respectively; Fig. 6d).

When the discrimination task became more difficult (odor pair 2), the mice in the DREADDs group were less successful at discriminating between the odors. Although there were no significant differences in individual blocks between the two groups, the mean number of blocks required to reach the 85% criterion was significantly higher for the DREADDs group than for the control group (the mean number of blocks to reach the 85% criterion was 13.6 for the DREADDs group and 10.1 for the control group, *n* = nine and 10 mice, respectively; [*t*(17) = 2.9, *p* = 0.01, unpaired *t*-test]; Fig. 6e). The difference became even more striking when the difficulty of the task was further increased with odor pair 3 (Fig. 6f). The ability of the mice in the DREADDs group to discriminate the odors in the odor pair was significantly lower than that of the mice in the control group. The number of blocks needed to reach the 85% criterion was significantly higher for the mice in the DREADDs group (the mean number of blocks to reach the 85% criterion was 16.3 and was obtained in a mean of 2.7 ± 0.2 days for the DREADDs group and 11.8 obtained in a mean of 2.2 ± 0.1 days for the control group, *n* = 10 and nine mice, respectively; [*t*(18) = 3.3, *p* = 0.01, unpaired *t*-test]; Fig. 6f), and significant differences in individual blocks were observed beginning from block 10 (Fig. 6f). These results show that CR^+^ GCs are involved in fine odor discrimination.

## Discussion

We morphologically and functionally characterized CR^+^ and CR^−^ GCs born at different developmental stages and used two different methods to show that CR^+^ neurons are involved in odor discrimination. First, an analysis of the expression of IEGs, cFos, and Zif268 following spontaneous and learned odor discrimination tasks showed that CR^+^ GCs are activated by these tasks. Second, the pharmacogenetic inhibition of CR^+^ CGs reduced the ability of the mice to discriminate between two similar odor mixtures. Our morpho-functional analysis of CR^+^ and CR^−^ GCs showed that there are fewer inhibitory synapses on the primary dendrites of CR^+^ GCs, which was corroborated by the lower frequencies of mIPSCs and sIPSCs recorded from this neuronal subtype. The reduced inhibition received by CR^+^ GCs may underlie the higher level of activation of this subtype following different odor discrimination tasks.

The inhibition provided by GCs in the OB synchronizes the activity of principal neurons, which is crucial for accomplishing different odor behaviors^37^. The involvement of CR^+^ GCs in odor discrimination remains unknown. Our observations showed that the pharmacogenetic inhibition of CR^+^ GCs reduces the fine discriminatory abilities of mice. It should be noted, however, that while the pharmacogenetic inhibition of CR^+^ GCs significantly reduced the ability of mice to discriminate between two similar odor mixtures, it did not abolish this ability completely. This can be explained by the fact that other GC subtypes also play a role in odor discrimination. For example, we recently showed that CaMKIIα^+^ GCs are involved in both spontaneous and go/no-go odor discrimination^38^. In addition, we estimate that about 8% of all CR^+^ GCs were infected with DREADDs, which may also explain why the odor discrimination ability was not abolished completely. Our findings are in line with a previous study showing that the optogenetic activation of comparable number of GCs is sufficient to affect odor discrimination^19^. Our results also do not rule out the involvement of superficial CR^−^ GCs in odor discrimination despite the fact that this subtype is less prone to activation during spontaneous and learned odor discrimination tasks. The use of pharmacogenetic or optogenetic approaches by injecting Cre-Off viral constructs into CR-Cre mice may help address this issue. CR is also expressed in the subpopulation of periglomerular interneurons^14,39^. However, our stereotaxic injections to pharmacogenetically inhibit CR^+^ cells in the OB were targeted to the GCL and resulted in very few virally-labeled CR^+^ periglomerular cells. It has been reported that periglomerular cells are involved in regulating theta, but not gamma, rhythms in the OB^4^, and that the odor discrimination abilities of mice depend on gamma oscillations^37^. These results suggest that the behavioral phenotypes observed following pharmacogenetic inhibition can be attributed to CR^+^ GCs but not CR^+^ periglomerular cells.

Our electrophysiological recordings showed that adult-born CR^+^ and CR^−^ GCs display similar sEPSC and mEPSC amplitudes and frequencies but distinct IPSC frequencies. The frequencies of sIPSCs and mIPSCs received by adult-born CR^+^ GCs were comparable to those of early-born CR^+^ GCs but lower than those of adult-born CR^−^ neurons at three and five wpi. Our results showing that there are fewer gephyrin^+^ puncta on CR^+^ GCs are in line with our electrophysiological results. The density of gephyrin^+^ puncta was lower on primary dendrites, but not on basal dendrites and cell soma, which are also targeted by inhibitory synapses in GCs^40–42^. Interestingly, olfactory learning affects the density of gephyrin^+^ puncta on the primary dendrites of adult-born GCs but not on basal dendrites or cell soma^5^. This indicates that inhibitory inputs to the primary dendrites of GCs may be particularly plastic and may be modified in response to the distinct sensory experiences of animals (olfactory learning) or different postsynaptic targets (CR^+^ or CR^−^ GCs). These results also suggest that CR^−^ and CR^+^ GCs may display different levels of inhibitory synapse plasticity following olfactory learning. It also remains to be determined whether these two subtypes of GCs receive inhibitory inputs originating from different sources or whether the presence of CR underlies the differences in the target cell-specific formation of inhibitory synapses.

Several studies have shown that early-born and adult-born GCs have different maturational profiles and undergo distinct odor experience-modulations^43–45^. Surprisingly, we observed no differences in the morphological or electrophysiological profiles of early- and adult-born CR^+^ GCs. It should be noted that comparisons of the morpho-functional properties of GCs born at different developmental stages have, until now, been performed on the entire GC population, without distinguishing between specific neurochemical subtypes^43–45^. However, GCs can be sub-divided into different subtypes with distinct temporal and spatial origins^21,46–48^. For example, very few CR^+^ GCs are produced during embryogenesis, and their production in the OB peaks at P30^21^, indicating that CR^+^ GCs make different contributions to the overall populations of early-born and adult-born neurons. Furthermore, early-born and adult-born GCs are preferentially located in the superficial and deep GCL, respectively^44,49^, and may thus display different synaptic input/output properties. It is thus possible that comparisons of the morpho-functional properties of the entire populations of early-born and adult-born GCs over-estimate differences due to different compositions of distinct neuronal subtypes in the populations of early-born and adult-born GCs and their localization in the GCL. Further studies will be required to determine whether and how early-born and adult-born GCs belonging to the same neuronal subtype differ in their morpho-functional properties and whether they are differentially involved in odor information processing. It has been also shown that among the adult-born population, the immature adult-born GCs are more prone to activation in response to the sensory input and display transient form of long-term potentiation at their proximal glutamatergic synapses^43,50^. It remains to be shown whether immature and mature adult-born CR^+^ GCs undergo distinct level of activation following habituation/dishabituation and go/no-go odor discrimination tasks.

Lastly, it should be noted that, in addition to CR^+^ GCs, several other GC subtypes have been identified based on the expression of mGluR2, CaMKIIα, CaMKIV, or glycoprotein 5T4^22–24^. We^38^ and others^24^ have described the roles played by CaMKIIα- and 5T4-expressing GCs in different odor behaviors. A deficiency in 5T4 affects the input/output properties of this subtype of GC, which has an impact on the odor detection threshold and on odor discrimination^24^. On the other hand, the expression of CaMKIIα defines functionally distinct subtypes of GCs involved in different odor behaviors. While CaMKIIα-expressing GCs are activated and are involved in the spontaneous and learned odor discrimination of structurally similar odors or odor mixtures, CaMKIIα-immunonegative GCs that receive higher levels of inhibition are involved in perceptual learning^38^. These studies, together with the results presented here, suggest that distinct GCs subtypes may be activated and involved in different odor tasks. Further studies will be required to determine the contributions of other GC subtypes to odor behavior.

Altogether, our results show that while the morpho-functional properties of early-born and adult-born CR^+^ GCs are indistinguishable, they receive lower levels of inhibition than adult-born CR^−^ neurons and are involved in the odor discrimination of chemically similar odors or odor mixtures.

## Material and Methods

### Animals

Experiments were performed using two- to four-month-old adult mice and postnatal 10-12-day-old (P10-P12) pups. The CalretininCre (CR-Cre; B6(Cg)-*Calb2*^*tm1(cre)Zjh*^/J; RRID:IMSR_JAX:010774)^51,52^ mouse line and the CalretininCre::CAG-CAT-GFP line obtained by cross-breeding CR-Cre and CAG-CAT-EGFP mice (FVB.B6-Tg(CAG-CAT-EGFP)1Rbns/KrnzJ; RRID:IMSR_JAX:024636)^53^ were used. Only hemizygous mice were used. The experimental procedures were in accordance with the guidelines of Canadian Council on Animal Care (CCAC), and all animal experiments were approved by the Université Laval animal protection committee. One to four mice per cage were kept on a 12-h light/dark cycle at a constant temperature (22 °C) with food and water *ad libitum*, except for the behavior experiments.

### Stereotaxic injections

The mice were anesthetized with an intraperitoneal injection of ketamine/xylazine (10 mg/mL and 1 mg/mL respectively; 0.1 mL per 10 g of body weight) and were kept on a heating pad during the surgery. Adeno-associated or lentiviral viral vectors (AAV and LV, respectively) were stereotaxically injected into the RMS or into the granule cell layer (GCL) of the OB. The following coordinates (with respect to the bregma) were used for the RMS injections in adult mice: anterior-posterior (AP) 2.55 mm, medio-lateral (ML) 0.82 mm, and dorso-ventral (DV) 3.15 mm; and for GCL injections: AP 4.6 mm, ML 0.75 mm, DV 1 mm and AP 5.3 mm, ML 0.5 mm, DV 0.9 mm. For injections in the RMS of CR-Cre pups, the following coordinates were used: AP 2.05 mm, ML 0.65 mm, and DV 2.7 mm. The following Cre-dependent viruses were used: rAAV 2/5 CAG-Flex-GFP (200 nL; 4 × 10^12^ iu/mL; Vector Core Facility, University of North Carolina), AAV 2/5-EF1α-DIO-EYFP (200 nL; 6.5 × 10^12^ iu/mL; Vector Core Facility, University of North Carolina), rAAV 2/8 EF1α-DIO-hM4D(G_i_)-mCherry (500 nL; 4 × 10^12^ iu/mL; University of North Carolina Vector Core Facility), and Cre-independent: AAV 2/5 miniCBA-Td-tomato (200 nL; 1.6 × 10^12^ GC/mL; Molecular Tools Platform, CERVO Brain Research Center), rAAV 2/5 CAG-GFP (100 nL; 4 × 10^12^ iu/mL; University of North Carolina Vector Core Facility), and lentiviral vectors encoding GFP expression (LV-GFP; 200 nL; 1.75 × 10^8^ tu/mL; University of North Carolina Vector Core Facility).

### Immunohistochemistry

The mice were given an overdose of pentobarbital (12 mg/mL; 0.1 mL per 10 g of body weight) and were perfused transcardiacally with 0.9% NaCl followed by 4% PFA. The OBs were resected and were post-fixed in 4% PFA at 4 °C. Horizontal sections (30- or 100-μm-thick) or coronal sections (40-μm-thick) were cut using a vibratome (Leica VT1000S) and were incubated with the following primary antibodies: mouse anti-GFP (48 h, 1:500, Invitrogen, Cat# A-11120, RRID:AB_221568), rabbit anti-mCherry (24 h, 1:1000, Biovision, Cat# 5993-100, RRID:AB_1975001), goat anti-calretinin (24 h, 1:1000, Millipore, Cat# AB 1550, RRID:AB_90764), rabbit anti-cFos (48 h, 1:40,000, Santa Cruz, Cat# Sc-52, RRID:AB_2106783), rabbit anti-Egr-1 (Zif268) (48 h, 1:150,000, Santa Cruz, Cat# Sc-110, RRID:AB_2097174), and anti-gephyrin fluorescently labeled with oyster-550 (24 h, 1:1000, Synaptic Systems, Cat# 147 011C3, RRID:AB_887716). The corresponding secondary antibodies were used. Fluorescent images were captured using a confocal microscope (FV 1000; Olympus) with 60× (UPlanSApoN 60×/NA 1.40 oil; Olympus) and 40× (UPlanSApoN 40× /NA 0.90; Olympus) objectives. Gephyrin images were captured using an LSM 700 AxioObserver microscope with a 63× objective (Plan-Apochromat 63×/NA 1.40 oil DIC M27, Zeiss). The images were captured from the GCs localized in the superficial GCL of the OB (up to 200 µm from the mitral cell layer). Coronal slices derived from the anterior, medial, and posterior OB were used for the IEG analysis, and horizontal slices derived from the dorsal, medial, and ventral OB were used for the morphological analysis. For the cFos analysis, three mice were used for both the habituation and dishabituation groups (spontaneous olfactory discrimination task), and three and five mice were used for the control and go/no-go groups, respectively. For the Zif268 analysis, four mice for used for both the habituation and dishabituation groups (spontaneous olfactory discrimination task), and three and five mice were used for the control and go/no-go groups, respectively.

### Patch-clamp recordings

Three to five weeks after the stereotaxic injections, the deeply anesthetized mice were transcardiacally perfused with modified oxygenated artificial cerebro-spinal fluid (ACSF) containing the following (in mM): 210.3 sucrose, 3 KCl, 2 CaCl_2_.2H_2_O, 1.3 MgCl_2_.6H_2_O, 26 NaHCO_3_, 1.25 NaH_2_PO_4_.H_2_O, and 20 glucose. The OBs were then quickly removed and were placed in oxygenated ACSF containing the following (in mM): 125 NaCl, 3 KCl, 2 CaCl_2_.2H_2_O, 1.3 MgCl_2_.6H_2_O, 26 NaHCO_3_, 1.25 NaH_2_PO_4_.H_2_O, and 20 glucose. Horizontal slices (250-μm-thick) were obtained using a vibratome (HM 650 V; Thermo Scientific). The slices were superfused with oxygenated ACSF at a rate of 2 mL/min during the electrophysiological experiments. Recordings were amplified using a Multiclamp 700B amplifier (Molecular Devices) and digitized using a Digidata 1440A (Molecular Devices).

Patch electrodes (ranging from 6 to 9 MΩ) were filled with intracellular solution containing the following (in mM): 135 CsCl, 10 HEPES, 0.2 EGTA, 2 ATP, 0.3 GTP, and 10 glucose for spontaneous inhibitory postsynaptic current (sIPSC) recordings or 130 K-methylsulfate, 10 HEPES, 6 KCl, 2 MgATP, 0.4 NaGTP, 10 Na-phosphocreatine, and 2 ascorbate for spontaneous excitatory postsynaptic current (EPSC) recordings. The intracellular solutions were also supplemented with 5 µM Alexa-Fluor 488 (Life Technology, A10436) or Alexa-Fluor 569 (Life Technology, A10438) to ensure that the recordings were taken from GFP or td-Tomato-infected cells.

sIPSCs were isolated by the bath application of kynurenic acid (5 mM Kyn; Sigma-Aldrich, Cat# K3375) to block glutamatergic activity. (−)-Bicuculline methochloride (50 μM BMI; Abcam Cat# AB120108), a GABA_A_ receptor antagonist, was used to isolate sEPSCs. Miniature IPSCs or EPSCs (mIPSCs or mEPSCs) were isolated by the application of tetrodotoxin (1 μM TTX; Affix Scientific, Cat# AF3015) in the presence of Kyn or BMI to block voltage-dependent sodium channels. Synaptic responses were analyzed using the MiniAnalysis program (Synaptosoft).

For electrophysiological recordings of CR^+^ GCs, two different Cre-dependent viruses (rAAV 2/5 CAG-Flex-GFP and AAV 2/5-EF1α-DIO-EYFP) were used. As expected, injection of these viral vectors into wild-type C57Bl/6 mice did not result in GFP expression. Surprisingly, however, CR immunostaining three weeks post-injection (wpi) of these AAVs in the RMS of CR-Cre mice revealed different levels of co-labeling for GFP and CR. While 98.0% of superficial GFP^+^ GCs were co-labeled for CR^+^ following the injection of AAV 2/5-EF1α-DIO-EYFP, only 48.6% of GFP^+^ GCs were labeled for CR following the injection of rAAV 2/5 CAG-Flex-GFP. The reason for the low percentage of GFP^+^/CR^+^ GC labeling following the injection of rAAV 2/5 CAG-Flex-GFP is unclear, but is likely related to different activities of the CAG and EF1α promoters during the maturation of neuronal precursors^54–56^. CR is transiently expressed during hippocampal and cortical development^52,57^ and in adult-born neuronal precursors in the dentate gyrus^58^. It is thus possible that the transient expression of CR in neuroblasts combined with the different levels of activities of the CAG and EF1α promoters drives GFP expression in a higher number of cells containing the CAG promoter than in cells containing the EF1α promoter. The same observations have been reported for the visual cortex, where cross-breeding CR-Cre mice with Cre-dependent CAG-tdTomato reporter mice results in the expression of tdTomato in cells that do not immunolabel for CR^52^. The functional responses of tdTomato^+^/CR^+^ and tdTomato^+^/CR^−^ neurons in the visual cortex were, however, indistinguishable^52^. In line with this, we did not observe any differences in the electrophysiological parameters of GCs infected with either the CAG-Flex-GFP or EF1α-DIO-EYFP viral vector and thus pooled the data.

To characterize the morpho-functional properties of CR^+^ and CR^−^ GCs, a mixture of Cre-dependent and Cre-independent AAVs driving GFP and tdTomato expression, respectively, were co-injected into the RMS of adult CR-Cre mice. To determine the specificity of CR^−^ GC infection by Cre-independent AAV, we quantified the percentage of tdTomato^+^/GFP^−^ GCs that were immunolabeled for CR. Our results revealed that 18.2 ± 1.5% of tdTomato^+^/GFP^−^ GCs were CR^+^. Hence, any difference in the morpho-functional properties observed using the two AAVs would be an underestimation given that CR^−^ GCs group was composed of approximately 20% CR^+^ GCs.

### Morphological analysis

The mice were sacrificed one, three, or five weeks after the viral vector injections into the RMS. Horizontal sections (100-μm-thick) of the OB were immunostained to reveal the presence of GFP, mCherry, and CR. Images were captured as described above. The images were zoomed four times for the spine analyses. The lengths of the primary dendrites were measured from the soma to the first branching point. The total lengths of the secondary dendrites were obtained by adding the lengths of all the individual secondary dendrites of a given cell. Basal dendrites were defined as dendrites beginning at the cell soma and extending in the opposite direction of the primary dendrite. The sum of all the basal dendrites was calculated for a given cell. Spine density was calculated by dividing the number of spines by the length of the section of the secondary dendrite. Adult-born CR^+^ and CR^−^ GC morphologies at the same wpi were obtained from the same slices, allowing for paired *t*-test comparisons.

### Analysis of gephyrin immunolabeling

The RMS of C57Bl/6 mice were injected with an AAV-GFP. The mice were sacrificed at three wpi. Horizontal slices (30-μm-thick) were obtained as described above and were immunolabeled for CR and gephyrin. Consecutive 0.35-μm-thick optical sections were analyzed using Fiji software. Median 3D filtering set at two was applied to the images. Gephyrin^+^ puncta were counted manually using the Cell Counter plug-in in Fiji. The dendritic length and the soma volume were measured using the Simple Neurite Tracer and 3D Object Counter plug-ins, respectively. The densities of gephyrin for CR^+^ and CR^–^ GCs were obtained from the same slices, allowing for paired t-test comparisons.

### Behavioral procedures

Seven to ten days before the behavioral experiments, individually housed male mice were put on a 12 h reverse light/dark cycle, with food and water *ad libitum*. The mice were sacrificed 1 h after the completion of each task to analyze the expression of cFos and Zif268 in the OB. The odors used were (−)-carvone (Sigma Aldrich, Cat# 22060), (+)-carvone (Sigma Aldrich, Cat# 22070), (+)-limonene (Sigma-Aldrich, Cat# 62118), (−)-limonene (Sigma-Aldrich, Cat# 62128), octyl aldehyde (octanal) (Sigma-Aldrich, Cat# 5608), and decanal (Sigma-Aldrich, Cat# 59581).

#### Odor discrimination

For the habituation/dishabituation odor discrimination task, the odors were presented in glass Pasteur pipettes containing filter paper soaked with 6 μL of 2% odorant in mineral oil. The odor presentations consisted of five min sessions at 20 min intervals. The mice were first accustomed to the glass pipette by presenting it to them twice. The mice were then subjected to four habituation sessions with a first odor ((+)-carvone). On the fifth session, one group of animals (control group) was subjected to the habituation odor, whereas a different group of animals (habituation/dishabituation group) was subjected to a new odor (dishabituation odor, (−)-carvone). The time spent exploring the pipette during each session was recorded.

#### Go/no-go olfactory discrimination learning

The mice were partially water-deprived until they reached 80 to 85% of their initial body weight before starting the go/no-go training. The animals were first trained to insert their snouts into the odor sampling port and to lick the water port to receive a 3 µl water reward. The reward-associated odor (S+) was then introduced. The mice initiated each trial by breaking the light beam across the odor sampling port, which opened an odor valve. The duration of the opening was increased progressively from 0.1 to 1 s. The mice with a minimum sampling time of 50 ms received a water reward. The mice usually successfully completed the training in one-two sessions. Following the training procedure, the mice were subjected to the go/no-go odor discrimination task. To ensure that the mice successfully learned the task, the first session consisted of 30 trials during which only the S+ odor was presented. Mice that reached at least an 80% success rate were then exposed randomly to a reward-associated odor (S+) or to a no reward-associated odor (S−) for several blocks of 20 trials each (random exposure to 10 S+ and 10 S−). Correct responses consisted of correct hits (mouse licking the water port after the S+ exposure) and correct rejections (mouse not licking the water port after the S− exposure). False responses consisted of the mouse licking the water port after the S− exposure or not licking the water port after the S+ exposure. The percentage of correct responses for each block of 20 trials was calculated. The mice were considered to have successfully completed the go/no-go olfactory discrimination task if they reached ≥ 85% of correct responses. The sessions lasted for one to five days, depending on the odorant pairs. The following three odor pairs were used: odor pair 1, 0.1% octanal (S+) *vs*. 0.1% decanal (S−) in 99.9% mineral oil; odor pair 2, 0.6% (+)- + 0.4% (−)-limonene (S+) *vs*. 0.4% (+)- + 0.6% (−)-limonene (S−) in 99% mineral oil; odor pair 3, 0.48% (+)- + 0.52% (−)-limonene (S+) *vs*. 0.52% (+)- + 0.48% (−)-limonene (S−) in 99% mineral oil. When the go/no-go olfactory discrimination task was coupled with the pharmacogenetic inhibition of CR^+^ GCs, the control and experimental groups were both subjected to the same behavioral protocol, with the same exposure to the S+ and S− odors. The only difference was that control group was injected with an AAV-GFP virus whereas the experimental group was injected with a DREADDs virus. To determine whether the go/no-go odor discrimination learning task induced subtype-specific activation of GCs, the mice in the experimental group were subjected to the protocol described above, whereas the mice in the control group were exposed equally and randomly to the two odors but received the water reward independently of the S+ or S− odor. The mice in the control group mice did not thus discriminate the odors. All the mice were sacrificed 1 h after completing the task and were immunolabeled for CR and cFos (or Zif268).

#### Pharmacogenetic inactivation of CR-expressing GCs

To study the role of CR^+^ GCs in the go/no-go olfactory discrimination task, we inactivated the CR^+^ GCs using the Designer Receptors Exclusively Activated by Designer Drugs (DREADDs) pharmacogenetic approach. We stereotaxically injected the Cre-dependent rAAV 2/8 EF1α-DIO-hM4D(G_i_)-mCherry viral vector bilaterally into the OB of CR-Cre mice and subjected them to go/no-go olfactory discrimination training two weeks after the injection. Following its expression, an evolved human M4 muscarinic receptor with two point mutations was activated by clozapine-N-oxide (CNO, 2 mg/kg, Tocris, Cat# 4936). This resulted in membrane hyperpolarization by the activation of an outward K^+^ current^36^. The control mice were injected with the rAAV 2/5 CAG-GFP viral vector. It has been shown *in vitro* and *in vivo* that the peak of CNO efficiency occurs after 20 min and last up to 180 min^59,60^. To estimate the efficacy of DREADDs virus, we injected Cre-dependent rAAV 2/8 EF1α-DIO-hM4D(G_i_)-mCherry virus (DREADDs group) and EF1α-DIO-EYFP virus injected (control group) into CR-Cre OB mice (*n* = three for each group). Two weeks after injection, animals received an intraperitoneal dose of CNO and were placed into clean individual cages for 90 min, followed by perfusion with 4% PFA. 40-μm-thick slices were cut and subjected to immunostainings against mCherry, GFP and cFos. The percentage of colocalization cFos^+^/DREADDs^+^ (DREADDs group) and cFos^+^/GFP^+^ (control group) were estimated. Our results demonstrate that DREADDs^+^ GCs are still inhibited at 90 min after CNO injection. For behavioral experiments, the two groups of mice received an intra-peritoneal injection of CNO every day, 25–30 min before starting the go/no-go task, which lasted approximately 45 min for each animal. No CNO was given during the training.

To estimate the percentage of DREADDs-infected CR^+^ GCs among the entire CR^+^ GC population, we first calculated the density of CR^+^ GCs in the GCL. Given that the viral vectors were injected into two sites of the GCL that covered the entire rostro-caudal portion of the dorsal OB, which makes up 50.0% of the entire GCL, and that 45.0% of CR^+^ GCs in the infected area were DREADDs^+^, we estimate that the percentage of DREADDs-infected CR^+^ GCs among the entire CR^+^ GC at approximately 8%.

### Statistical analysis

Male and female mice were used for all the experiments, except the behavior experiments, which were performed only with males. Data are expressed as means ± SE. Statistical significance was determined using a one-way ANOVA test followed by a Bonferroni post hoc test or a paired or unpaired two-sided Student’s *t*-test, depending on the experiment, or a non-parametric Mann-Whitney *U* test as indicated in the text and tables. For the morphometric and gephyrin immunolabelling experiments, we first analyzed 7–12 CR^+^ and CR^−^ GCs in the same animal to obtain the average values for these two subtypes. We then used an ANOVA or paired Student’s *t*-test to assess the level of significance across the animals. The exact value of *n* and its representation (cells, animals) is indicated in the text and corresponding tables. No statistical methods were used to predetermine the sample size. Equality of variance for the unpaired *t*-test was verified using the F-test. The levels of significance were as follows: *p < 0.05, **p < 0.01, ***p < 0.001. When possible, the investigator was blinded to the experimental conditions (all the IEG experiments and gephyrin analysis). The data that support the findings of this study are available upon request.
